# Low-Frequency Sound Absorption Mechanism and Bidirectional Prediction of a Viscoelastic Rubber-Based Underwater Acoustic Coating Using Multimodal Deep Ensemble Learning

**DOI:** 10.3390/polym18060693

**Published:** 2026-03-12

**Authors:** Zhihao Zhang, Renchuan Ye, Nianru Liu, Guoliang Zhu

**Affiliations:** Ocean College, Jiangsu University of Science and Technology, Zhenjiang 212003, China

**Keywords:** acoustic coating micro-perforated panel, transfer matrix method, sound absorption coefficient

## Abstract

Underwater acoustic coatings are widely used to suppress low-frequency noise radiation and sonar reflection in underwater vehicles. In this study, an underwater acoustic coating model consisting of viscoelastic rubber layers and micro-perforated panel (MPP) structures is investigated, with particular emphasis on the low-frequency sound absorption mechanism and predictive modeling. Based on an improved transfer function method, a novel Micro-Perforated Panel Acoustic Coating Layer (MPPACL) model is developed to describe the coupled acoustic behavior of multilayer coatings under underwater conditions. The low-frequency sound absorption performance is primarily governed by the viscoelastic characteristics of the rubber layer, including material damping and complex modulus, while the incorporation of the MPP further enhances absorption through resonance effects. To efficiently explore the relationship between structural parameters and acoustic response, an ensemble learning-based deep neural network (ELDNN) is constructed using analytically generated data, enabling both forward prediction of sound absorption performance and inverse prediction of structural design parameters. The results show that the frequency prediction accuracy of the IDNN model is 3.7 times that of the DNN model. Furthermore, the proposed MPPACL model has achieved a significantly enhanced sound absorption effect within the frequency range of 50 to 2000 hertz. This effect has also been further verified through underwater experiments. The proposed framework provides an efficient and reliable approach for the design and optimization of underwater acoustic coatings.

## 1. Introduction

Noise reduction research has become a crucial global challenge in modern industry and daily environments. The rapid development of engineering fields such as ships [1,2], and submersible vehicle [3] has raised higher demands for noise control technology, especially in the area of low-frequency noise management. However, due to the long-wave nature of low-frequency sound waves [4], existing technologies face numerous challenges in effectively absorbing low-frequency noise.

In the study of acoustic cover layers, various methods have been employed to evaluate the sound absorption performance of different materials and their underwater applications. First, resonance and wave propagation methods are used to measure the complex Young’s modulus of highly compliant elastomers, discussing the impact of transverse inertia on measurements [5]. Then, elastic theory and transfer function methods are applied to analyze the noise reduction characteristics of underwater acoustic damping materials, validate the effect of high-frequency rubber fillers, and explore the sound reflection properties of different composite materials at low frequencies [6,7]. Dynamic viscoelastic performance measurement methods are used to evaluate the temperature and nonlinear effects of materials, and the Young’s modulus, Poisson’s ratio, and damping factors of silicone rubber are determined through laser triangulation and finite element methods [8,9]. In the study of noise-reducing coatings with steel plate substrates, finite element analysis reveals the sound absorption peak caused by resonance between the steel plate and the rubber layer, and discusses the impact of coating thickness [10,11]. Additionally, a liquid–solid cooperative sound absorption mechanism is employed to develop ultra-thin soundproof coatings, which convert longitudinal waves into shear waves in the viscous liquid and bending waves in the viscoelastic solid to attenuate sound waves, solving the issue of low-frequency broadband absorption [12,13]. Non-invasive stochastic modeling methods are used to study the reflection and transmission loss of submarine sound-absorbing coatings, optimizing coating geometry and physical parameters to enhance acoustic stealth [14]. Based on split-hole resonators and optimized air cavity technology, a broadband sound absorption system suitable for deep-sea environments has been developed to support submarine stealth technology [15,16,17]. Furthermore, a new cavity structure is proposed based on low-frequency absorption and air cavity enhancement technology, aimed at enhancing broadband sound absorption capacity in deep-water regions [18]. Additionally, the liquid–solid cooperative sound absorption mechanism has been employed to design a 3 mm ultra-thin coating, successfully achieving low-frequency broadband sound absorption in the 1–1000 Hz frequency range, resolving the conflict between coating thinning and low-frequency absorption [19].

In the study of MPP, various theoretical and experimental methods have been used to analyze their vibration and sound absorption characteristics. First, the virtual mass effect of fluid on the plate is analyzed to assess changes in natural frequencies and modes, with the added virtual mass increment calculated using Rayleigh-Ritz and Green’s function methods [20,21,22]. System resonance modes are used to describe the time response of fluid-loaded structures, and numerical and experimental validations are conducted to determine resonance frequencies and the required number of modes [23]. For underwater porous plate structures, the acoustic impedance is obtained based on air porous plate absorption theory, and sound transmission characteristics are analyzed using the transfer matrix method, calculating the vertical incidence transmission coefficient, with wave tube experiments for verification [24]. The free vibration of circular plates in immersed fluid is analyzed by least squares, Galerkin and Rayleigh-Ritz methods, addressing fluid–structure coupling and singularities, with comparisons to experimental and finite element results [25]. Applying the low-frequency sound absorption advantages of micro-perforated panel structures to underwater acoustic coatings, provides new ideas for developing deep-water pressure-resistant sound absorption technology and breaking through the current mid-low frequency and broadband sound absorption bottlenecks of underwater vehicles. As an important fundamental sound absorption structure in the field of acoustics, micro-perforated panels exhibit excellent low-frequency sound absorption characteristics under limited space constraints [26,27].

In recent years, artificial intelligence technologies, especially deep learning models, have shown great potential in the field of materials and structural design due to their powerful nonlinear modeling capabilities. Huang [28,29,30,31] et al. proposed a Helmholtz resonator with embedded hole structures, optimized for sound absorption performance using genetic algorithms, achieving a sound absorption coefficient above 0.9 in the 280–480 Hz range through the “weak coupling” effect between different cavities. Zhenqian [32] used deep learning to construct an auto encoder network to solve the inverse design problem of metasurfaces, optimizing 9 resonators and achieving broadband low-frequency sound absorption with a sound absorption coefficient above 0.9 in the 350–530 Hz range. BaoZhu Cheng [33] used MLP and an optimized DAE model for acoustic structure inverse design, improving DAE prediction accuracy by 2.64 times, with a center frequency deviation of only 9 Hz. Yiping Sun [34] proposed a topology optimization method based on variational autoencoders, constructing a dataset using the finite element method, training the VAE model to generate new types of sound-absorbing coatings, and quickly generating high-performance designs for efficient optimization. Gao [35,36] used the transfer matrix method and CNN models to achieve high-precision inversion design, significantly shortening the design cycle for complex acoustic structures. Mahesh, K [37] proposed an inverse design method based on deep neural networks, creating datasets through theoretical methods to predict the geometric parameters of Helmholtz resonators and micro-perforated panels, achieving absorber design optimization. Deep learning technology can quickly predict sound absorption performance through a data-driven approach, providing a new solution for the optimized design of broadband sound absorption performance of parallel micro-perforated panels. The use of deep learning not only significantly improves design efficiency but also allows the exploration of complex design spaces that traditional design methods cannot cover, providing theoretical and technical support for innovations in sound-absorbing materials and structures.

This paper mainly focuses on two aspects of research: the application of the proposed MPPACL model in underwater sound absorption, and the acoustic prediction capability of the improved ELDNN model applied to the MPPACL model. Based on an improved transfer function method, a theoretical model is proposed and verified. Through this model, the sound absorption advantages of microperforated panels in the low-frequency band are analyzed, which shows significantly enhanced sound absorption performance especially in the frequency range of 50 Hz to 2000 Hz. Subsequently, for this model, ensemble learning combined with deep neural network is used to conduct forward and inverse prediction on its characteristic parameters, and the integration of attention mechanism further enhances the ability to process complex data. Finally, based on a comparative analysis of the two methods, the key parameters affecting sound absorption performance are identified, the design of the acoustic coatings is optimized, and a theoretical basis is provided for the improvement of underwater sound absorption technology. The core innovation lies in proposing a deep ensemble model (ELDNN) that integrates “structure–acoustics” dual-modal features, which extracts key features within modalities through deep sub-networks and improves the bidirectional prediction accuracy of low-frequency sound absorption performance and structural parameters.

## 2. Theoretical Framework

### 2.1. Theoretical Model

**Figure 1:**
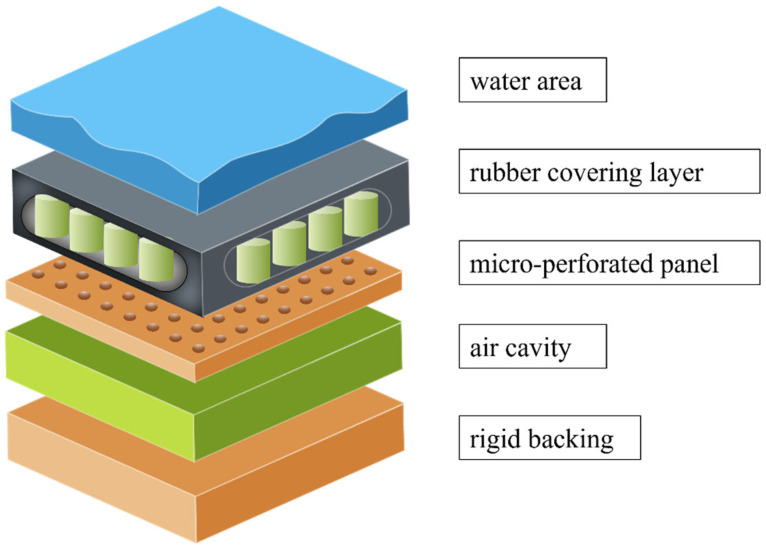
Schematic diagram of the MPPACL theoretical model.

In this section, a theoretical model of the micro-perforated panel acoustic coating layer (MPPACL), as shown in Figure 1, is proposed. The model combines the propagation characteristics of axisymmetric acoustic waves in cylindrical tubes with a layered modeling strategy for acoustic attenuation layers with gradually varying axial pores, which significantly reduces the computational complexity of the multilayer acoustic system while maintaining sufficient prediction accuracy. For the two structures coupled in the present model, the viscoelastic rubber layer mainly dissipates acoustic energy through its inherent material damping [13]. When the incident sound wave induces structural vibration, the internal friction within the rubber matrix converts part of the mechanical energy into heat, leading to energy attenuation. In addition, the impedance mismatch between the rubber layer and the surrounding medium also causes partial reflection and additional energy dissipation.

For the micro-perforated panel (MPP) [24], the sound absorption effect is mainly governed by viscous and thermal losses inside the micro-scale perforations. When acoustic waves pass through these small holes, the oscillating airflow experiences strong viscous resistance along the hole walls and thermal exchange with the solid boundaries, which converts acoustic energy into heat. This mechanism produces a resonance-type sound absorption behavior when the acoustic impedance of the perforated structure matches that of the surrounding medium. When the viscoelastic rubber layer is combined with the micro-perforated panel structure, these two mechanisms interact with each other, providing both broadband damping and enhanced resonant absorption, thereby improving the overall sound absorption performance of the composite acoustic structure.

To quantitatively describe the acoustic behavior of the multilayer structure, the homogenization method is first used to calculate the effective acoustic impedance of each layer by deriving macroscopic acoustic parameters from the internal microstructure of the materials. For rubber layers and perforated rubber layers, variations in porosity and medium fillers are considered, and the acoustic properties of gradient structures with different porosities and fillings are described through volume fraction correction. Subsequently, the transfer matrix method (TMM) is applied to calculate the propagation process of sound waves through the multilayer structure and their attenuation effects. For perforated structures, a modified Maa model is adopted to evaluate the acoustic impedance of the micro-perforated panels, in which both viscous losses and end correction mass effects are taken into account. Finally, by cascading the transfer matrices of all layers, the overall acoustic response of the system is obtained, and the total reflection coefficient, as well as the sound absorption coefficient, are calculated to evaluate the acoustic performance of the attenuation layer.

### 2.2. Theoretical Calculation

#### 2.2.1. Equivalent Rubber Layer Matrix

In the simplified theory, a one-dimensional acoustic wave propagation model is employed to describe the acoustic behavior of each layer, neglecting tangential effects, with state variables defined as:

(1) $$s(z)=\left[\begin{array}{c} p(z)\\ v(z) \end{array}\right]$$

where *p*(*z*) represents the acoustic pressure (Pa), and *v*(z) denotes the normal particle velocity (m/s). For any equivalent fluid-type sublayer with thickness *d*, its state satisfies the following transfer relationship between the upper and lower surfaces [12]:

(2) $$s(0)=Ts(d),T_{fluid(kz,Z_{e},d)}=\left[\begin{array}{cc} \cos(k_{c}d) & iZ_{c}\sin(k_{c}d)\\ \frac{i}{Z_{c}}\sin(k_{c}d) & \cos(k_{c}d) \end{array}\right]$$

where *k_z_* = (*ω*/*c*) cos *θ* is the normal wavenumber, *ω* = 2π*f* is the angular frequency (rad/s), *Z_c_* = *ρc*/cos *θ* is the characteristic impedance of the layer (Pa·s/m), *ρ* is the medium density (kg/m^3^), *c* is the medium sound speed (m/s), and *θ* is the incidence angle.

For pure rubber layers, the longitudinal wave equivalent fluid approximation is employed, and the longitudinal wave sound velocity is calculated through Lamé constants (*λ*, *μ*) [12]:

(3) $$\lambda =\frac{vE}{\left(1+v)(1-2v\right)},\mu =\frac{E}{2(1+v)},c_{0}=\sqrt{\frac{E(1-v)}{\rho_{r}(1+v)(1-2v)}},Z_{0}=\frac{\rho_{r}c_{0}}{\cos\theta }$$

where *E* is the complex modulus of rubber (Pa), *v* is the Poisson’s ratio of rubber, *ρ* is the density of rubber (kg/m^3^), *C*_0_ is the longitudinal wave velocity (m/s), and *Z*_0_ is the characteristic impedance of rubber (Pa·s/m).

**Figure 2:**
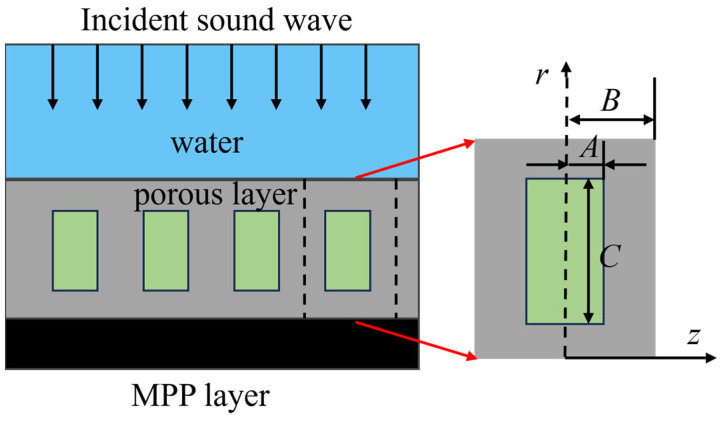
Cross-sectional view of the acoustic coating model.

As shown in Figure 2, for perforated rubber layers, the volume fraction method is used to modify the density and wavenumber [12]:

(4) $$\rho_{\mathrm{eff}}=(1-\xi )\rho_{r}+\xi \rho_{c},\xi =\pi {\left(\frac{A}{B}\right)}^{2},k_{z}=\frac{\omega }{c_{l}}\cos\theta ,c_{l}=\sqrt{\frac{\lambda +2\mu }{\rho_{\mathrm{eff}}}}$$

where *A* is the pore radius (m), *B* is the matrix radius (m), *ξ* is the volume fraction, and *P_c_* is the density of the pore-filling medium (kg/m^3^).

With each layer thickness being *L*1, the transfer matrices are multiplied layer by layer to obtain:

(5) $$T_{\mathrm{rubber}}=T_{0}\times \prod_{i=1}^{6}T_{i}\times T_{2}$$

*T*_0_ represents the transfer matrix of the top pure rubber layer. This matrix describes the propagation process of sound waves as they enter the top pure rubber layer from the external medium, including the propagation, attenuation, and reflection characteristics of sound waves within this layer.

*T_i_* represents the transfer matrix of the rubber layer containing air columns. *T_i_* is used to describe the propagation characteristics of sound waves through the intermediate layer, encompassing acoustic behaviors such as absorption and reflection. Each intermediate layer, depending on its structure and material properties, has a corresponding transfer matrix *T_i_*.

*T*_2_ represents the transfer matrix of the bottom pure rubber layer. Similarly to *T*_0_, *T*_2_ describes the process of sound waves propagating from the bottom pure rubber layer to the underlying medium.

#### 2.2.2. Microperforated Panel Matrix

**Figure 3:**
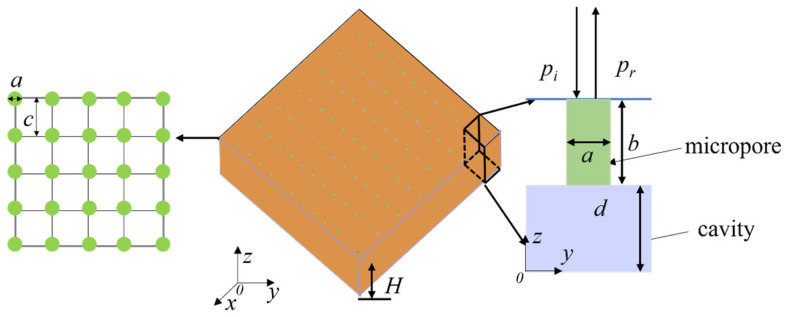
Structural diagram of the micro-perforated panel absorber.

As shown in Figure 3, a microperforated absorber consisting of multiple Helmholtz resonators is considered, where the number of holes forms an *m* × *n* matrix. All Helmholtz resonators within the matrix share identical geometry. The overall thickness of the absorber structure is *H*, with the upper portion comprising small holes less than 1 mm in diameter and the lower portion consisting of a cavity. The holes have a diameter a and thickness *b*, while the cavity has a thickness *d*. Since the holes are arranged periodically relative to the plate structure, it can be understood as circular holes of diameter a embedded in a square grid with side length *c*. This configuration simplifies the understanding of porosity. In the modeling, the acoustic impedance of the micro-perforated panel can be represented as [21]:

(6) $$Z_{1}=\frac{32\eta b}{a^{2}}{\left(1+\frac{k^{2}}{32}\right)}^{1/2}+j\omega \rho_{0}b(1+{\left(32+\frac{k^{2}}{2}\right)}^{-1/2})$$

where *ρ* represents the air density, *η* represents the air viscosity coefficient, *a* represents the diameter of the holes, and *b* represents the thickness of the holes. $k=\frac{a}{2}\sqrt{\rho_{0}\omega /\eta },\;\;\;1<k<10$, *k* represents the ratio of the radius to the boundary layer thickness.

The relative acoustic impedance of the perforated panel is given by [21]:

(7) $$Z_{\mathrm{total}}=Z_{1}/(\sigma \rho_{0}c)=r+j\omega_{m}$$

where *σ* represents the porosity, $r=\frac{32\eta b}{\sigma \rho_{0}c_{0}a^{2}}[{\left(1+\frac{k^{2}}{32}\right)}^{1/2}+\frac{\sqrt{2}}{32}k\frac{a}{b}]$,

$$\omega_{m}=\frac{\omega t}{\sigma c_{0}}[1+{\left(9+\frac{k^{2}}{2}\right)}^{-1/2}]+0.85\frac{a}{b}$$

When sound waves propagate through the micro-perforated panel, the absence of transverse wave transmission results in the following transfer matrix for the overall rigid micro-perforated structure:

(8) $$T_{MPP}=\left[\begin{array}{cc} 1 & Z_{\mathrm{total}}\\ 0 & 1 \end{array}\right]$$

The material parameters of the overall structure are listed in Table 1.

#### 2.2.3. Sound Absorption Coefficient Calculation

The total transfer matrix is obtained by cascading the transfer matrices of each individual layer through multiplication:

(9) $$T_{\mathrm{tot}}=T_{\mathrm{rubber}}\cdot T_{\mathrm{mpp}}\cdot T_{\mathrm{cavity}}\cdot T_{\mathrm{plate}}$$

*T_rubber_* represents the transfer matrix of the rubber layer, which characterizes the propagation, reflection, and absorption characteristics of sound waves within the rubber material.

*T_mpp_* denotes the transfer matrix of MPP layer, which primarily influences the low-frequency sound absorption performance, describing the acoustic behavior of the micro-perforated panel.

*T_cavity_* corresponds to the transfer matrix of the cavity layer, which governs the propagation and attenuation characteristics of sound waves within the cavity, often influenced by resonance effects and the geometry of the cavity. The cavity matrix calculated by Equation (5).

*T_plate_* represents the transfer matrix of the plate layer, typically approximated using the mass law, and describes the reflection and transmission effects of the plate on sound waves.

*T_plate_*, steel plate, approximated using the mass law:

(10) $$Z_{\mathrm{plate}}=i\omega \rho_{s}h_{s}$$

System input impedance:

(11) $$Z_{\mathrm{in}}=\frac{AZ_{L}+B}{CZ_{L}+D}$$

*A* and *D* are the coefficients related to the reflection and transmission of sound waves within the system. *B* and *C* are the coefficients that describe the propagation of sound waves and the coupling effects between the different layers.

Reflection coefficient and sound absorption coefficient:

(12) $$R=\frac{Z_{\mathrm{in}}-Z_{w}}{Z_{\mathrm{in}}+Z_{w}},\;\alpha =1-|R{|}^{2}$$

### 2.3. Simulation Validation

**Figure 4:**
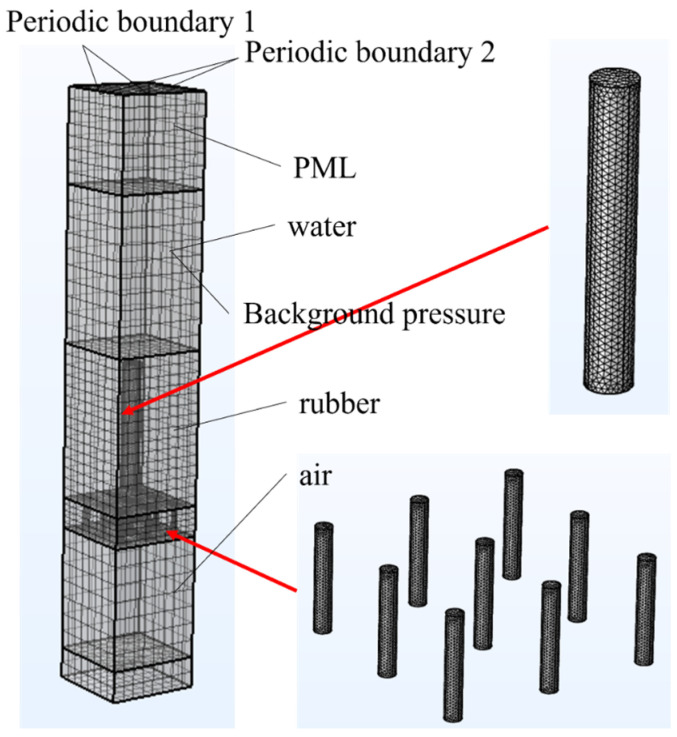
Schematic diagram of the MPPACL simulation model.

To verify this theoretical model, we conducted a finite element simulation using COMSOL 6.2. As shown in Figure 4, leveraging the periodic nature of the structure, a single unit cell was selected for modeling. A swept mesh was employed for the structural components, while “free triangular” elements were used to discretize the cavity domain, resulting in a total of 245,003 elements. In the physics settings, the “Acoustic-Structure Interaction” module was applied. Specifically, the rubber domain was simulated using the “Elastic Waves” interface, the air within the pores and the external water domain were modeled with the “Pressure Acoustics” interface, and the micro-perforated panel’s air domains were specially configured using the “Narrow Region Acoustics” feature.

**Figure 5:**
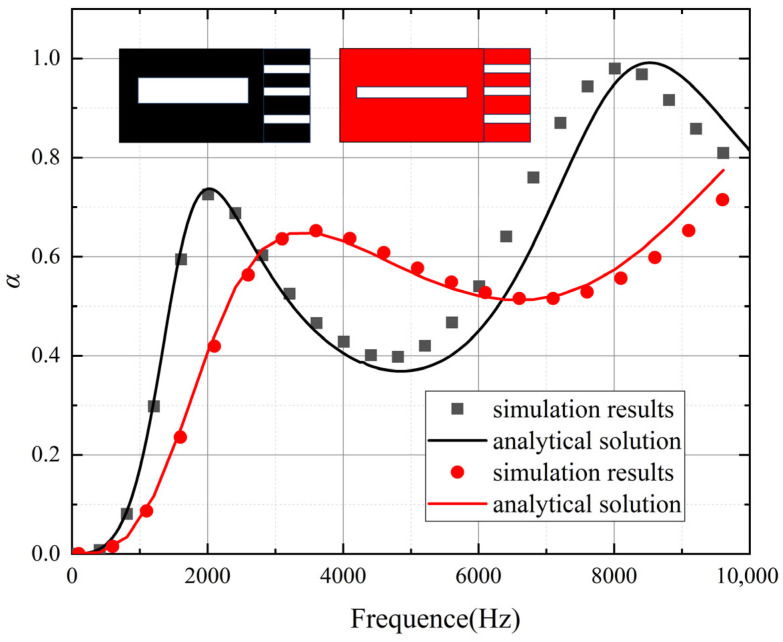
Comparison of sound absorption coefficients.

As illustrated in Figure 4, both the acoustic coating and the micro-perforated panel employ typical cylindrical pores for computation. In this study, focusing on representative underwater applications, the thickness of the acoustic attenuation layer is fixed at 50 mm, with pore height set to 40 mm and pore radius maintained at 1 mm. Each unit cell is designed as a square with a side length of 15 mm. The micro-perforated panel has a fixed thickness of 10 mm with pore radii of 0.8 mm. The computational results were validated against analytical solutions, while comparative analysis was conducted between two structures with pore diameters of 1 mm and 3 mm within the acoustic coating. As shown in Figure 5, the results demonstrate excellent agreement, confirming the accuracy of both the theoretical derivation and numerical simulations presented in this work.

### 2.4. Effect of Micro-Perforated Structures on Acoustic Coating Performance

**Figure 6:**
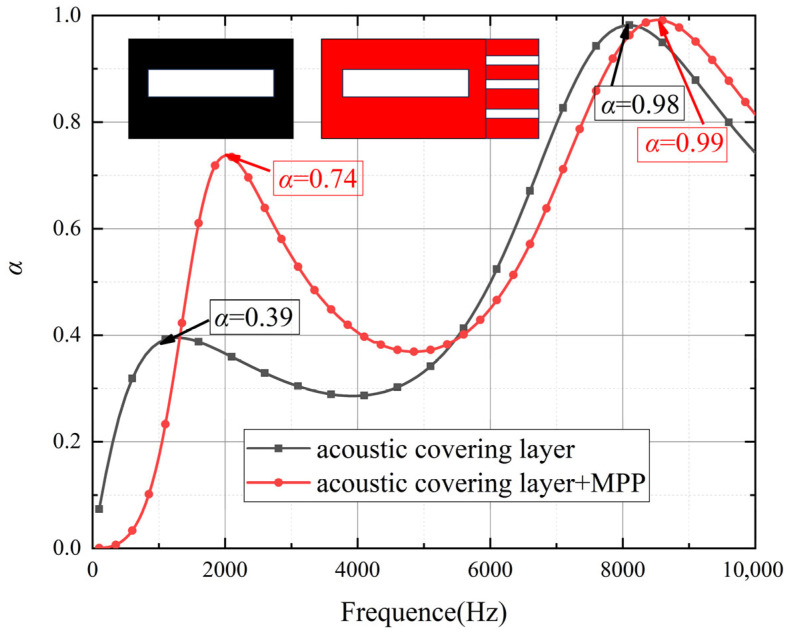
Sound absorption coefficient curves of micro-perforated panels with different aperture sizes.

**Figure 7:**
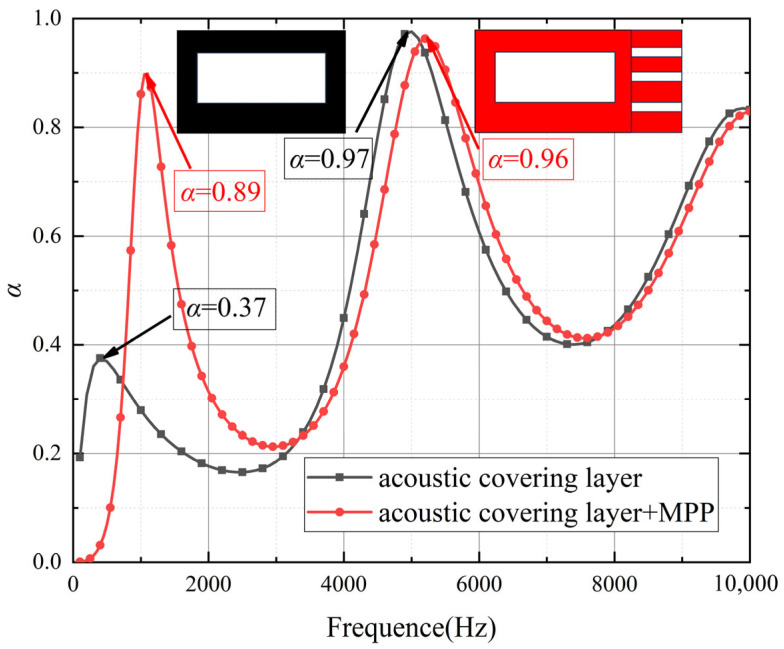
Comparison of sound absorption performance between micro-perforated panel and non-micro-perforated panel structures.

**Figure 8:**
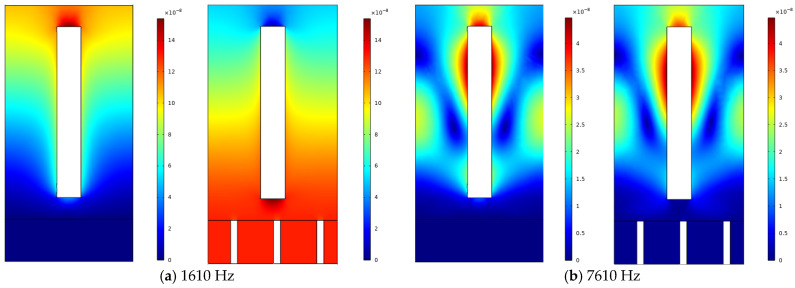
Comparison of displacement contour plots at 1610 Hz and 7610 Hz with a 3 mm aperture.

**Figure 9:**
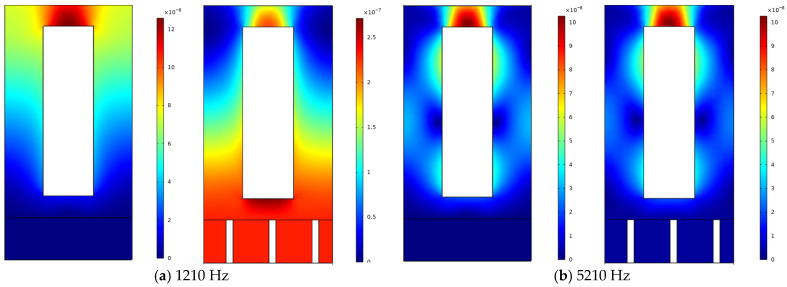
Comparison of displacement contour plots at 1610 Hz and 7610 Hz with a 6 mm aperture.

To validate the advantages of the proposed model for low-frequency sound absorption in acoustic coatings, this section compares the acoustic performance of coatings with and without MPP across different frequency ranges. As shown in Figure 6 and Figure 7, the curves indicate that the structure incorporating MPP significantly outperforms the non-MPP structure in low-frequency sound absorption. In the frequency range of 50 Hz to 2000 Hz, the MPP-based acoustic coating exhibits higher sound absorption coefficients, with a distinct peak observed around 1600 Hz, demonstrating its effectiveness in absorbing low-frequency acoustic energy. This enhanced performance can be attributed to the resonance effects between the micro-perforations and the cavity behind the MPP, which efficiently dissipate sound energy, particularly in the low-frequency range. As shown in Figure 8 and Figure 9, The geometric configuration of the micro-perforations, when matched to the wavelength of the incident sound, leads to increased interaction and energy dissipation, resulting in higher absorption [24]. In contrast, the coating without MPP shows lower absorption coefficients in the same frequency range, indicating inferior performance due to the absence of such resonance mechanisms. In the high-frequency range (2000 Hz to 8000 Hz), the sound absorption of the MPP-integrated structure shows no significant improvement compared to the non-MPP structure. Even at higher frequencies, the absorption coefficients of the MPP and non-MPP configurations remain comparable, suggesting limited enhancement in high-frequency absorption. This limitation is due to the reduced interaction between high-frequency sound waves and the micro-perforation structure, as the wavelength of high-frequency waves becomes much smaller than the perforation size, thus diminishing the effectiveness of the resonance mechanism. Consequently, MPP is less effective in absorbing higher-frequency waves, which require a different structural configuration to achieve optimal absorption performance.

## 3. Deep Learning Framework and Methods

### 3.1. Database Establishment

In the construction of deep learning models, data quality and diversity are crucial. To investigate the multi-frequency sound absorption performance of the MPPACL, this study established a ‘structure–acoustic’ bimodal dataset covering a wide range of parameters. This database essentially represents the organic integration of two types of modal data: Structural Modal Data: Includes geometric parameters of different MPPACLs (acoustic coating radius A, micro-perforated panel radius a, porosity δ) and material properties (rubber density, elastic modulus, etc.), reflecting the physical morphological characteristics of the sound-absorbing structure. Acoustic Modal Data: Includes sound absorption performance parameters of the structure across different frequency bands (frequency *f*, sound absorption coefficient *α*), reflecting the structure’s response characteristics to sound waves.

These two types of modal data were generated synchronously via analytical solutions, ensuring parameter correspondence and laying the foundation for subsequent multimodal feature fusion. Specifically, this paper constructed a three-dimensional acoustic performance database using three geometric parameters—the acoustic coating radius A, the micro-perforated panel radius a, and the porosity δ—forming a dataset with a broad value range. For subsequent analytical convenience, these three parameters were redefined as parameters a, b, and c.

The variation ranges and step sizes for these parameters are as follows: Parameter a ranges from 1 × 10^−3^ to 16 × 10^−3^ with a step size of 0.2 × 10^−3^, resulting in 76 nodes; Parameter c ranges from 0.1 × 10^−3^ to 1 × 10^−3^ with a step size of 0.05 × 10^−3^, resulting in 19 nodes; Parameter d ranges from 1 to 16 with a step size of 1, resulting in 16 nodes. Through combinations of these parameters, a total of 76 × 19 × 16 = 23,104 data groups were generated. To enhance the quality and effectiveness of the database, we performed further statistics: all data fall within the 200–4000 Hz range and have a sound absorption coefficient greater than 0.4; within this, data in the low-frequency band (50–2000 Hz) account for 65%, highly aligning with the core research focus on low-frequency sound absorption scenarios in this paper and ensuring the multimodal model’s learning effectiveness for key frequency bands. These data not only cover a vast parameter space but also ensure a high proportion of high sound absorption performance data, greatly enhancing the model training effectiveness.

### 3.2. ELDNN Model

**Figure 10:**
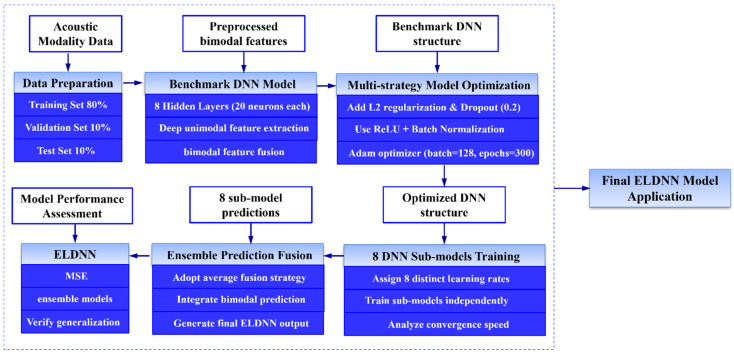
ELDNN Model Flowchart.

In this paper, we propose an Ensemble Learning-based Deep Neural Network (ELDNN) model that enhances predictive performance by integrating multiple Deep Neural Network (DNN) sub-models. Initially, we designed a benchmark DNN model comprising an input layer, 8 deep hidden layers (each containing 20 neurons), and an output layer. The first 4 hidden layers are used to extract deep unimodal features (the structural modality extracts associative features among geometric parameters, the acoustic modality extracts response features between frequency and sound absorption coefficient), while the last 4 layers achieve the concatenation and fusion of bimodal features, replacing traditional shallow ensemble strategies like simple voting. The design of the benchmark model laid the foundation for subsequent model optimization and improvement. The specific process is shown in Figure 10.

Building upon this, we introduced multiple optimization strategies to enhance model performance. Firstly, adjusting the learning rate was one of our primary optimization methods. By finely tuning the learning rate, we could control the model’s convergence speed, avoiding oscillations caused by an excessively large learning rate or slow convergence due to an overly small one. Secondly, to reduce overfitting, we incorporated L2 regularization and dropout techniques. L2 regularization prevents the model from overfitting to noise during training by adding a squared penalty term for parameters to the loss function, while dropout enhances model robustness during training by randomly dropping a portion of neurons. Furthermore, we optimized the activation function, selecting the ReLU (Rectified Linear Unit) activation function and introducing Batch Normalization technology. This helps reduce training bias caused by distribution differences in the ‘structure–acoustic’ bimodal features, further enhancing the deep model’s ability to fit multimodal features and its computational efficiency. The specific parameters are shown in Table 2.

**Figure 11:**
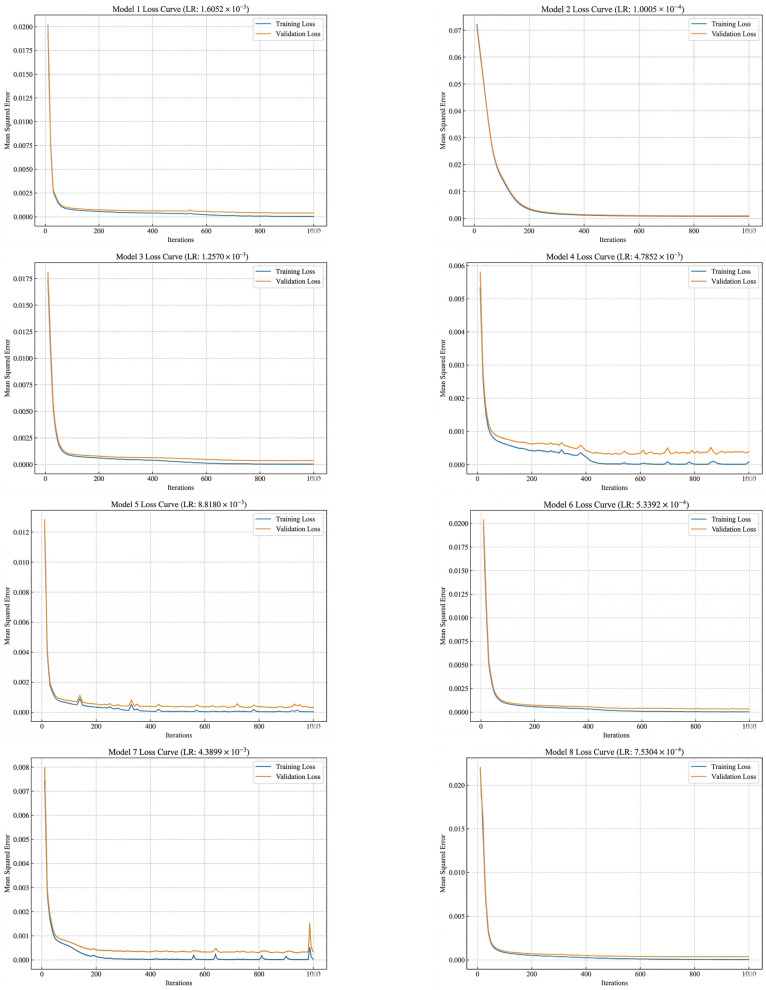
Loss Function Graphs of the 8 Models.

During the design process of the ELDNN model, we integrated eight improved DNN sub-models, each optimized to some extent based on the benchmark model. We selected different learning rates for each model (ranging from 1.0005 × 10^−4^ to 8.8180 × 10^−3^) and observed the impact of these different learning rates on the training and validation loss curves. From the loss curve plots, it can be seen that as the learning rate was adjusted, the training and validation losses of the models exhibited different convergence characteristics. Specifically: Model 1 (Figure 11) used a relatively large learning rate (1.6052 × 10^−3^). Both training and validation losses decreased rapidly and converged quickly to low values. However, the validation loss showed significant fluctuations in some iterations, indicating that the larger learning rate might lead to instability during convergence. Model 2 (Figure 11) used a smaller learning rate (1.0005 × 10^−4^). Its training process was smoother, with smaller fluctuations in validation loss, but the convergence speed was slower, suggesting that a smaller learning rate provides a more stable training process. Model 3 (Figure 11) used a medium learning rate (1.2570 × 10^−3^). Both training and validation losses showed good convergence, and the model stability was good, with a relatively smooth validation loss curve. Model 4 (Figure 11) used a larger learning rate (4.7852 × 10^−3^). The training loss decreased rapidly, but the validation loss exhibited obvious fluctuations, indicating that while a larger learning rate accelerates convergence, it might cause the model to over-adjust in some cases, affecting its stability. Model 5 (Figure 11) accelerated the training process with a high learning rate (8.8180 × 10^−3^), but its validation loss showed severe fluctuations in the later stages of training, suggesting that a high learning rate might lead to premature convergence without reaching the optimal solution. Model 6 (Figure 11) used an even smaller learning rate (5.3392 × 10^−4^). Its loss curves were relatively smooth, and the training and validation set losses were close, demonstrating strong stability. Model 7 (Figure 11) used a medium learning rate (4.3899 × 10^−3^). The training loss decreased rapidly, and the validation loss stabilized, showing good convergence characteristics. Model 8 (Figure 11) used a small learning rate (7.5304 × 10^−4^). Its training process was stable, the loss curve was close to the benchmark model, and the validation loss also showed good stability.

To further evaluate the performance of the improved ELDNN model, we compared it with the benchmark model using Mean Squared Error as the loss function for evaluation. By comparing the loss curves of different sub-models, we found that all improved models significantly reduced both training and validation losses, and the validation loss values were small, indicating that these optimization strategies effectively enhanced the model’s generalization ability. Particularly during training, the loss curves of multiple models showed rapid convergence, indicating that the introduced optimization methods (such as learning rate adjustment, regularization, and activation function selection) effectively prevented overfitting while accelerating model convergence. Furthermore, the lower validation losses indicate that the model can achieve good predictive performance on unseen data, thereby enhancing its practical application value. Through this ensemble-optimized ELDNN model, we have not only improved the stability and accuracy of the DNN model but also provided an effective solution for complex prediction tasks.

**Figure 12:**
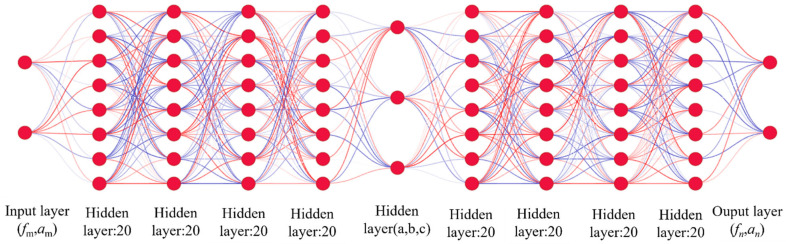
Architecture of the ELDNN Model.

As depicted in the Figure 12, each network is composed of an input layer, an output layer, and several hidden layers. The weights of the hidden layers are set during the training phase. Using the described theoretical method, we derived the performance parameters *f* and *α* corresponding to these randomly chosen geometric parameters.

## 4. Ensemble Learning Prediction Results and Discussion

### 4.1. Acoustic Performance Prediction Within the Dataset

To enhance the persuasiveness of the prediction results, this section divides the dataset into three groups: one comprising 80% of the data is used as the training data for prediction, while the other two groups, each containing 10% of the data, are used as in-dataset prediction data and out-of-dataset prediction data, respectively. Specifically, 24 sets of data are selected as the target parameters for prediction. Sets 1–12 are chosen in ascending order of frequency, while sets 13–24 are randomly selected from the dataset, ensuring the diversity of the prediction data. As shown in Figure 11, the prepared parameters are input into the ensemble model ELDNN for prediction. Since all 24 target parameters are derived from the database, this is referred to as in-dataset acoustic performance prediction.

In terms of prediction, defining the three-dimensional physical parameters (a, b, c) as the input for ELDNN model training and the two-dimensional parameters (f, α) as the output is termed forward prediction. Conversely, defining the two-dimensional performance parameters (f, α) as the input and the three-dimensional geometric parameters (a, b, c) as the output is termed backward prediction. These two prediction approaches allow for the prediction of sound absorption coefficient metrics based on model parameters, while also enabling the structural design of the model based on desired target performance.

Analyzing from the perspective of “structure–acoustic” bimodal correlation (combined with data from Table 3), when the structural modal parameter a (aperture) falls within the range of 0.0028–0.0068 m, the prediction deviation for the acoustic modal parameter f (frequency) is minimized (≤25 Hz), and the prediction deviation for α is ≤0.005. This indicates that within this aperture range, the structural and acoustic modalities exhibit the highest degree of matching—a synergistic relationship that is difficult to capture with traditional single-modal models, thereby validating the enhancement of prediction accuracy through multimodal feature fusion.

To improve the accuracy of the results, the outcomes of the eight models were computed independently and then averaged, yielding the final prediction results presented in this paper.

(13) $$\bar{m}=\frac{\sum_{i=1}^{8}x_{i}}{8}$$

As shown in Table 3 and Table 4, the maximum deviation of parameter *a* is 0.000071, the minimum deviation is 0.00003, and the average deviation is 0.000027. For parameter *b*, the maximum deviation is 0.0016, the minimum deviation is 0.000053, and the average deviation is 0.000324. For parameters *c*, the maximum deviation is 0.0011, the minimum deviation is 0.0001, and the average deviation is 0.000306. From the prediction results, the ELDNN model demonstrates good accuracy, with the predicted results nearly equal to the actual data from the analytical solutions.

In Table 3, the maximum deviation of frequency f is 51 Hz and the minimum deviation is 9 Hz; the maximum deviation of the sound absorption coefficient α is 0.0291 and the minimum deviation is 0.0003; the maximum deviation of geometric parameter a is 0.001174 m and the minimum deviation is 0.000009 m; the maximum deviation of b is 0.000071 m and the minimum deviation is 0.000007 m; the maximum deviation of c is 0.7743 m and the minimum deviation is 0.1184 m. In Table 4, the maximum deviation of *f* is 43 Hz and the minimum deviation is 2 Hz; the maximum deviation of α is 0.0109 and the minimum deviation is 0.0005; the maximum deviation of a is 0.000237 m and the minimum deviation is 0.000012 m; the maximum deviation of b is 0.00006 m and the minimum deviation is 0.00001 m; the maximum deviation of c is 2.0729 m and the minimum deviation is 0.3147 m.

**Figure 13:**
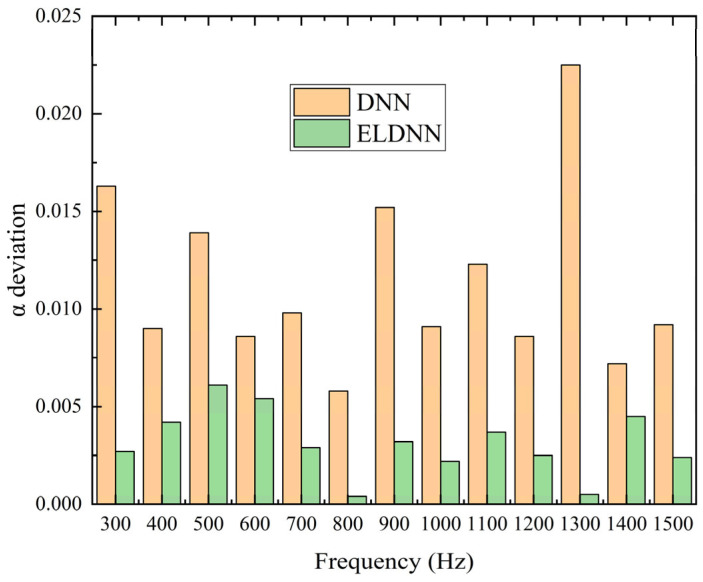
Comparison of Frequency and Absorption Coefficient Deviations in Prediction Results.

As shown in Figure 13, the ELDNN model proposed in this paper demonstrates better prediction performance than the conventional DNN model for the peak absorption coefficient α over the entire frequency range. For the DNN model, the deviation of α varies between 0.0058 and 0.0224, with an average deviation of approximately 0.011. In contrast, the ELDNN model shows significantly smaller deviations, ranging from 0.0004 to 0.0061, with an average deviation of about 0.003. Compared with the DNN model, the proposed ELDNN model reduces the average deviation of the peak absorption coefficient by about 3.7 times, indicating that the proposed model can significantly improve the prediction accuracy and stability. The specific accuracy results are shown in Table 5.

### 4.2. Acoustic Performance Prediction Outside the Dataset

In Table 6, the maximum deviation of frequency f is 63 Hz and the minimum deviation is 2 Hz; the maximum deviation of the sound absorption coefficient α is 0.0142 and the minimum deviation is 0.0001; the maximum deviation of geometric parameter a is 0.000682 m and the minimum deviation is 0.000017 m; the maximum deviation of b is 0.000029 m and the minimum deviation is 0.000007 m; the maximum deviation of c is 0.9937 m and the minimum deviation is 0.2656 m. In Table 7, the maximum deviation of f is 41 Hz and the minimum deviation is 4 Hz; the maximum deviation of α is 0.0087 and the minimum deviation is 0.0002; the maximum deviation of a is 0.000162 m and the minimum deviation is 0.000012 m; the maximum deviation of b is 0.000066 m and the minimum deviation is 0.000019 m; the maximum deviation of c is 1.0029 m and the minimum deviation is 0.3018 m.

**Figure 14:**
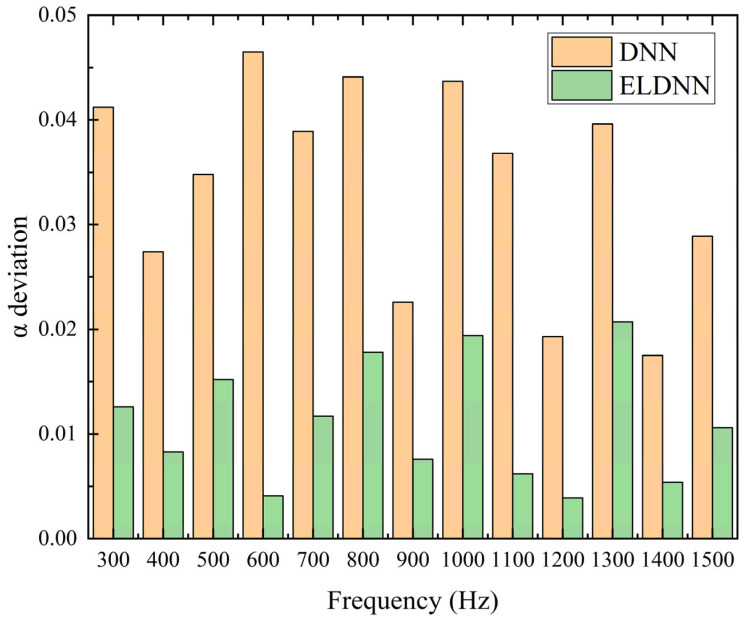
Comparison of Frequency and Absorption Coefficient Deviations in Prediction Results.

As shown in Figure 14 and Table 8, the deviations of the peak absorption coefficient predicted by the DNN and ELDNN models at different frequencies are compared. It can be observed that the ELDNN model consistently produces smaller deviations than the DNN model across the entire frequency range from 300 Hz to 1500 Hz. For the DNN model, the deviation ranges approximately from 0.017 to 0.046, with an average deviation of about 0.034. In contrast, the ELDNN model shows significantly smaller deviations, varying between 0.004 and 0.021, with an average deviation of about 0.011. Compared with the DNN model, the ELDNN model reduces the average deviation of the peak absorption coefficient by about three times, demonstrating that the proposed model provides more accurate and stable prediction performance. In summary, the IDNN model excels over the DNN model in predicting both frequency and absorption coefficient. However, the DNN model’s accuracy decreases for parameters outside the dataset.

### 4.3. Effect of Micro-Perforated Panel Parameters on Sound Absorption

**Figure 15:**
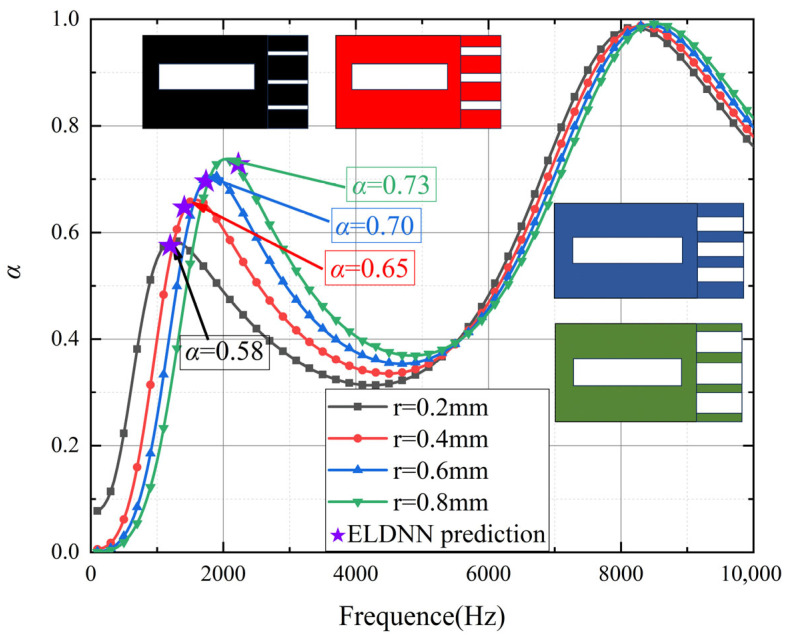
Effect of pore size variations in micro-perforated panels on sound absorption coefficient.

As shown in Figure 15 Effect of pore size variations in micro-perforated panels on sound absorption coefficient, the influence of different aperture sizes in micro-perforated panels on the sound absorption coefficient varies with frequency. The figure displays the sound absorption coefficients of micro-perforated panels with aperture sizes of 0.2 mm, 0.4 mm, 0.6 mm, and 0.8 mm across different frequencies. In the low-frequency range of 0–2000 Hz, the sound absorption coefficient increases with larger aperture sizes. Particularly at 1750 Hz, the panel with a 0.2 mm aperture exhibits a relatively low absorption coefficient of approximately 0.58, while the panel with a 0.8 mm aperture achieves a higher absorption coefficient of nearly 0.73. These results indicate that micro-perforated panels with larger apertures more effectively absorb acoustic energy in the low-frequency range. This enhanced performance can be attributed to the resonance effect between the sound waves and the apertures. Larger apertures, with their increased size, allow for greater interaction with the longer wavelengths of low-frequency sound waves, leading to more effective absorption. Smaller apertures, on the other hand, may allow stronger sound wave transmission due to their reduced size, which limits the resonance effect and thus inhibits low-frequency absorption performance. The gradual improvement in the absorption coefficient with increasing aperture size suggests that larger apertures facilitate better resonance and energy dissipation in the low-frequency range, where the wavelengths of sound are longer and more compatible with the aperture dimensions. However, in the high-frequency range of 2000–10,000 Hz, the influence of aperture size on the sound absorption coefficient gradually diminishes, and the absorption coefficients for all aperture sizes converge. This indicates that at higher frequencies, variations in aperture size have a limited impact on sound wave absorption, with all apertures exhibiting similar absorption effects. This reduction in the influence of the micro-perforated structure at high frequencies is likely due to the shorter wavelengths of high-frequency sound waves, which are less efficiently absorbed by the micro-perforated panels because the smaller apertures are no longer well-matched to the shorter wavelengths, reducing the resonance effect.

At 1750 Hz, the displacement contour plots of micro-perforated panels with different aperture sizes show minimal differences. The observations indicate that the displacement distribution and vibration patterns of the four aperture sizes are fundamentally similar at this frequency, with consistently small vibration amplitudes. This suggests that, regardless of aperture size variations, all panels effectively suppress vibrations and exhibit comparable vibrational responses at 1750 Hz. The similarity in displacement contours implies that, while aperture size influences low-frequency sound absorption performance, the vibration suppression effects across the four aperture variations are nearly identical at this specific frequency, with no significant distinctions. This phenomenon can be attributed to the fact that, at 1750 Hz, the apertures are sufficiently large to allow for effective interaction with the sound waves, which in turn reduces vibration amplitudes. Beyond this point, further increases in aperture size have diminishing returns on vibrational behavior, as the panels reach an optimal size where the vibrations are already effectively dampened, and additional increases in aperture size do not significantly enhance vibration reduction.

Furthermore, the prediction results using the ELDNN (Ensemble Learning-based Deep Neural Network) method are also shown in the figure (purple stars). By training the deep learning model, it becomes possible to more efficiently predict the sound absorption coefficients of micro-perforated panels with different aperture sizes across various frequencies. Compared to traditional experimental data, the ELDNN model provides a data-driven approach capable of autonomously learning and predicting sound absorption performance under different aperture conditions. To train the model, we used experimental data as input, incorporating factors such as the aperture of the micro-perforated panel, material properties, and frequency. The trained ELDNN model can achieve high-precision predictions of sound absorption coefficients across different frequency ranges.

In the low-frequency range, the sound absorption coefficients predicted by ELDNN show high consistency with experimental results. The ELDNN model can accurately predict the peak sound absorption coefficients for different aperture sizes. Compared to traditional theoretical models and experimental data, the deep learning approach, through training on large datasets and modeling nonlinear relationships, can more precisely capture the sound absorption characteristics of micro-perforated panels, especially in complex frequency responses. The advantage of this method lies in its ability not only to compensate for the limitations of experimental data but also to effectively process and predict details that are difficult to measure during experiments.

As shown in Figure 15, the porosity of micro-perforated panels significantly influences their sound absorption performance and vibration control characteristics. The absorption coefficient curves demonstrate that reducing porosity substantially diminishes the acoustic absorption effectiveness. At a porosity of 0.0804, the absorption coefficient reaches 0.82 in the low-frequency range, indicating exceptional low-frequency absorption capability. As porosity decreases, the absorption coefficients progressively decline to 0.73 at a porosity of 0.0201, 0.57 at 0.0050, and 0.45 at 0.0013. These results clearly demonstrate that higher porosity values enhance low-frequency sound absorption performance, as larger pores allow for greater energy dissipation through the resonance effect between sound waves and the micro-perforated structure. Conversely, lower porosity values result in smaller pores, which reduce the interaction between sound waves and the panel, limiting the resonance effect and thus significantly compromising absorption capacity, particularly in the low-frequency domain. This is because, in the low-frequency range, sound waves with longer wavelengths are more effectively absorbed by panels with larger pores that better match the wavelengths of the sound waves, allowing for more efficient sound energy dissipation.

**Figure 16:**
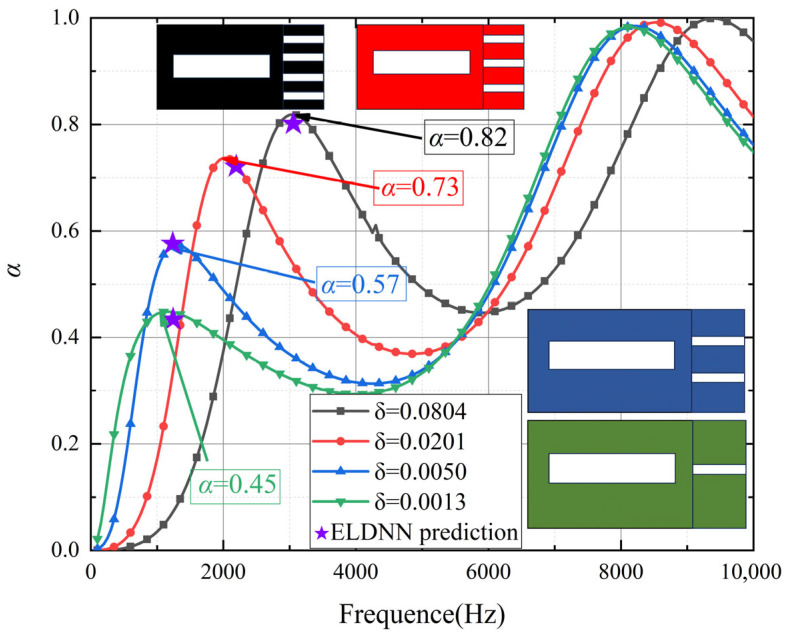
Effect of porosity variations in micro-perforated panels on sound absorption coefficient.

As shown in Figure 16, the four displacement contour plots demonstrate minimal variations despite the gradual decrease in porosity from 0.0804 to 0.0013. Across all cases, the micro-perforated panels exhibit similar vibration patterns, characterized by relatively small vibrations around the pores and a generally uniform distribution of vibration amplitudes. This observation indicates that the vibration control effectiveness of the micro-perforated panels remains at a relatively stable level at 1750 Hz, regardless of porosity variations. The consistency in vibration patterns across different porosity levels suggests that, at this specific frequency, the micro-perforated structure has already reached an optimal state for vibration suppression. This phenomenon may be attributed to the fact that, at 1750 Hz, the panel’s structure, particularly the perforation size and distribution, is well-suited to dampen vibrations efficiently, and further reducing porosity does not significantly alter the vibrational behavior. The relatively stable vibration response across different porosities suggests that the vibration control mechanism is governed more by the overall structure of the micro-perforated panel than by the porosity alone.

Although micro-perforated panels with higher porosity demonstrate superior low-frequency sound absorption performance in the absorption coefficient curves, the displacement contour plots reveal that reduced porosity has limited impact on vibration amplitude. This suggests that, at this specific frequency, porosity exerts less influence on vibration control compared to its effect on sound absorption coefficients. The absence of significant variations in displacement contours may be attributed to the fact that, at the specific frequency considered, vibration control mechanisms are primarily governed by the overall structural design and material properties of the micro-perforated panel, such as perforation size, distribution, and the mechanical properties of the base material, rather than porosity alone. While porosity enhances sound absorption by influencing the resonance characteristics of the panel, vibration control appears to be more dependent on the structural integrity and stiffness of the material, which determines the panel’s ability to suppress vibrations effectively. Therefore, even with a reduction in porosity, the structural design remains a key factor in vibration suppression performance.

### 4.4. Influence of Rubber Coating Parameters on Sound Absorption

**Figure 17:**
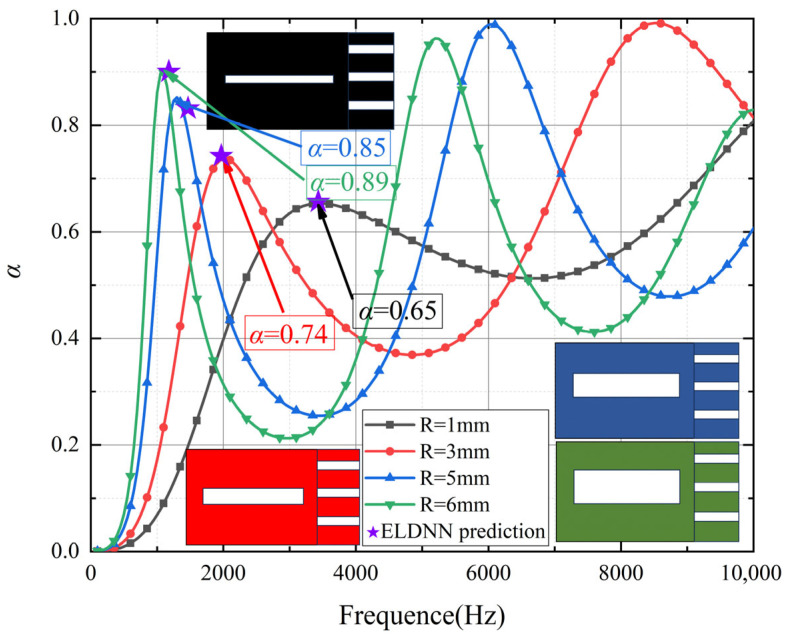
Effect of acoustic coating aperture size on sound absorption coefficient.

As shown in Figure 17 Effect of acoustic coating aperture size on sound absorption coefficient, increasing the aperture size of the acoustic coating significantly enhances its low-frequency sound absorption capability. At 1000 Hz, the coating with a 3 mm aperture demonstrates the highest absorption coefficient of 0.75, while smaller apertures achieve only 0.55 and 0.58. This indicates that larger apertures facilitate more effective interaction with low-frequency sound waves, promoting wave penetration and absorption, thereby improving low-frequency performance. The improved interaction between sound waves and the coating’s structure can be attributed to the fact that larger apertures allow for more efficient resonance with low-frequency sound waves, increasing energy dissipation. However, further increasing the aperture to 4 mm leads to saturation in absorption improvement. At 1000 Hz, the 4 mm aperture achieves an absorption coefficient of 0.78, showing negligible difference compared to the 3 mm aperture (0.75). This suggests that excessively large apertures may reduce the system’s damping capacity, leading to diminishing returns in low-frequency absorption. When apertures become too large, the coating loses its ability to efficiently absorb sound energy, as the larger gaps reduce the resonance effect that enhances absorption. At higher frequencies (2000 Hz and 3500 Hz), the absorption coefficients of all aperture sizes converge to approximately 0.65 and 0.60, respectively, indicating reduced sensitivity to aperture variations and stabilized absorption performance in the high-frequency regime. This behavior can be explained by the fact that at higher frequencies, the sound wavelengths become shorter and are less effectively coupled with the larger apertures, leading to a more uniform absorption performance across different aperture sizes.

Analysis of the displacement contour plots reveals that aperture variations exert distinct effects on vibration patterns in low and high frequency ranges. At 1000 Hz, the coating with a 3 mm aperture demonstrates a smaller maximum displacement of approximately 0.12 mm, while the 0.5 mm aperture exhibits a larger vibrational response with 0.18 mm maximum displacement. The larger aperture effectively reduces low-frequency vibrations by enhancing the resonance interaction between the sound waves and the perforated panel, which leads to more efficient vibration damping and improved acoustic absorption performance. In contrast, smaller apertures, which provide less opportunity for resonance with low-frequency sound waves, result in larger vibrations. As frequency increases to 1250 Hz, 2000 Hz, and 3500 Hz, displacement differences gradually diminish across all aperture sizes. Particularly at 3500 Hz, the maximum displacements converge to approximately 0.1 mm regardless of aperture size, indicating minimal aperture influence on high-frequency vibrations. This behavior suggests that at higher frequencies, the wavelength of the sound waves becomes smaller, and their interaction with the aperture structure becomes less significant. At high frequencies, the absorption and vibration suppression effects are more influenced by the intrinsic eigenmodes of the panel-cavity system, with aperture size playing a reduced role. These results demonstrate that increased aperture size significantly improves low-frequency vibration control but has limited impact on high-frequency vibrations. In the high-frequency regime, the absorption performance is predominantly governed by the panel-cavity resonance modes, and aperture effects become progressively less influential.

### 4.5. Comparison with Existing Methods

To further evaluate the effectiveness of the proposed ELDNN framework, a comparison with existing prediction and optimization methods reported in the literature was conducted. Traditional approaches for acoustic structure design mainly rely on numerical simulations combined with parametric sweeps or optimization algorithms. Although these methods can provide accurate results, they typically require a large number of repeated simulations, which significantly increases computational cost.

Machine learning methods have recently been introduced to accelerate acoustic performance prediction. For example, regression-based models and conventional deep neural networks (DNN) have been applied to approximate the nonlinear relationship between geometric parameters and acoustic performance. However, these methods often suffer from limited prediction stability and reduced accuracy when dealing with complex parameter spaces.

Compared with these existing methods, the proposed ELDNN model integrates ensemble learning with deep neural networks, which improves prediction robustness and reduces the influence of individual model bias. The statistical results presented in Table 2, Table 3, Table 4 and Table 5 show that the ELDNN model achieves lower prediction errors than the conventional DNN model. In particular, the average deviation of the absorption coefficient is reduced from approximately 0.011 to about 0.003, representing an improvement of nearly three times in prediction accuracy.

Furthermore, once the ELDNN model is trained, the prediction of structural parameters corresponding to target acoustic performance can be obtained almost instantaneously, while traditional optimization methods may require hundreds or even thousands of simulation iterations. Therefore, the proposed ELDNN framework provides an efficient and reliable tool for the inverse design of acoustic structures.

### 4.6. Computational Efficiency Analysis

The computational cost of the proposed ELDNN framework is relatively low compared with traditional simulation-based optimization approaches. In conventional acoustic structure design, parameter optimization typically relies on repeated numerical simulations, such as parametric sweeps or evolutionary algorithms. These approaches require a large number of acoustic simulations, which significantly increases computational time.

In contrast, the proposed ELDNN method only requires computational effort during the training stage. Once the model is trained, the prediction of structural parameters corresponding to target acoustic performance can be obtained through a simple forward propagation process in the neural network. Since the network architecture contains only five hidden layers with 20 neurons per layer, the computational complexity of the prediction process is very low. As a result, the prediction time for a single sample is typically on the order of milliseconds on a standard workstation.

Therefore, compared with traditional simulation-based optimization methods that may require hundreds of repeated simulations, the proposed ELDNN framework significantly reduces computational cost and enables rapid inverse design of acoustic structures.

## 5. Experimental Validation of Sound Absorption Structure

The previous computational results have validated the accuracy and efficiency of the proposed ensemble model. Next, we will experimentally verify its effectiveness in practical applications. Based on the geometric parameters predicted by the ensemble model in Table 1. During the fabrication process, the rubber layer containing the internal pores was prepared using customized molds. The mold cavities were designed according to the predicted pore diameter and spacing, ensuring that the geometric parameters of the pores could be controlled with reasonable accuracy. Liquid rubber material was poured into the mold and allowed to cure under controlled conditions, forming the porous rubber layer after demolding. This process ensured that the internal pore structures were formed according to the designed geometric configuration.

The micro-perforated plate was manufactured using 3D printing technology. This method allows precise control of the perforation diameter, hole distribution, and structural thickness. After printing, the plate was carefully cleaned to remove residual material and ensure that the perforations remained unobstructed. The fabricated micro-perforated plate was then aligned with the rubber layer according to the designed structural configuration. To facilitate fabrication and assembly, the entire structure was divided into three separate components during the manufacturing process. These components were subsequently assembled and bonded together using adhesive to form the complete acoustic structure. During assembly, particular attention was paid to maintaining the alignment of the perforations and ensuring that the interfaces between components were properly sealed. Finally, waterproof treatment was applied to the assembled sample to prevent water infiltration into the internal cavities during the experimental measurements. The completed structure was then installed in the experimental setup for acoustic testing.

**Figure 18:**
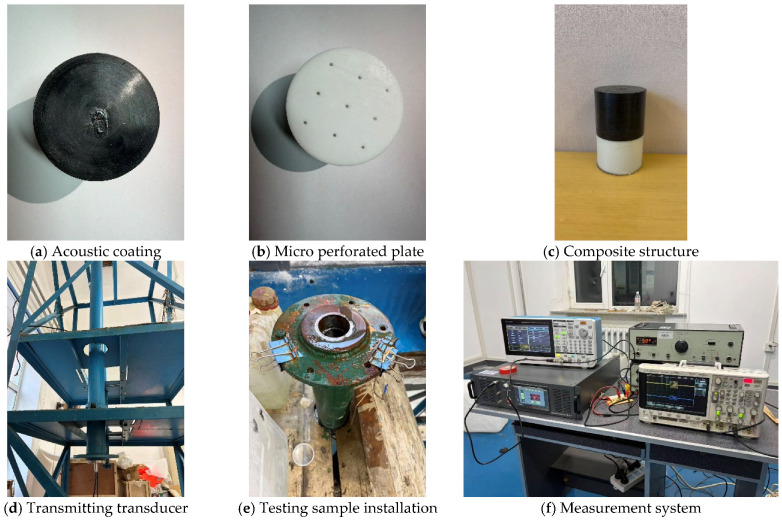
Sample and experimental process display diagram.

In this experiment, the pulse-tube measurement technique was employed. The acoustic tube device used in the experiment is a water-filled rigid thick-walled steel tube. A perfect reflector was placed at the upper end of the acoustic tube, and a rigid backing was selected according to the experimental requirements. Meanwhile, the lower end of the tube is closed and equipped with a transmit-receive integrated transducer. The acoustic tube has a length of 4 m, an inner diameter of 57 mm, and an outer diameter of 116 mm, and is placed vertically as a whole. Acoustic isolation was applied between the transmit-receive integrated transducer and the steel tube to reduce the excitation of tube wall vibration. The transducer was excited in an axisymmetric manner to maximize the upper frequency limit of the pulse acoustic tube, and the amplitude distribution on the radiation surface was kept as uniform as possible to minimize the amplitude of axisymmetric higher-order waves. For the backing, an acoustically hard end was used as the backing condition—specifically, a steel block was placed at the end of the acoustic tube to simulate an acoustically hard boundary. The equipment connection is shown in Figure 18f. Due to experimental constraints, the frequency points measured in this experiment were selected within the range of 5 kHz to 10 kHz.

The workflow of the entire experimental system is as follows: First, a signal source emits a pulsed sinusoidal signal with a specific amplitude, frequency, and pulse width. After being amplified by a power amplifier, the signal drives the transmit-receive integrated transducer to emit sound waves. These sound waves then propagate through the acoustic tube, and after a certain period of time, the transmit-receive integrated transducer receives the echo signal. The echo signal is first amplified by the input terminal of a measurement amplifier, then filtered, and after filtering, it is amplified again by the output terminal of the measurement amplifier. Finally, the amplified and filtered echo signal is transmitted to an oscilloscope, which displays the echo waveform.

**Figure 19:**
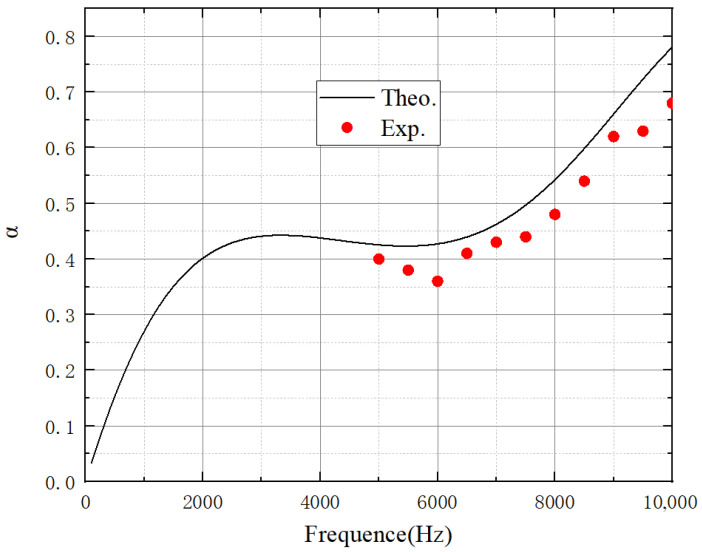
Comparison of experimental validation.

Considering factors related to experimentation and manufacturing, a set of geometric parameters outside the dataset was selected for comparison: a = 1 mm, b = 0.8 mm, c = 1. As shown in Figure 19, according to the comparison between the measured and predicted curves, the maximum deviation of the absorption coefficient is approximately 0.68, which occurs at around 6000 Hz, while the average deviation over the entire frequency range remains within 0.5. The experimental results were slightly lower than the analytical results, which can be attributed to several sources of error. First, the rubber material is relatively soft, leading to changes in the pore diameter during fabrication and installation. Slight deformation of the rubber structure may also occur under acoustic excitation, which can influence the effective acoustic impedance of the structure. Second, during the experiment, the system was not left to stand for a sufficiently long time, resulting in air bubbles remaining in the acoustic tube—this also affected the experimental outcomes. In addition, small deviations in the geometric parameters during manufacturing, such as variations in perforation diameter and cavity depth, may lead to differences between the theoretical model and the actual sample. The impedance tube measurement system itself may also introduce minor uncertainties due to sensor calibration accuracy, acoustic leakage at the tube interfaces, and environmental factors such as temperature and humidity variations. However, when examining the overall curve trend, the experimental results show a high degree of proximity to the predicted results. This further verifies the reliability of the ELDNN model in the inverse design of acoustic structures.

## 6. Discussion

The results presented in this study demonstrate the effectiveness of the proposed ELDNN model for predicting acoustic performance and performing inverse design of micro-perforated acoustic coating structures. Beyond the prediction accuracy demonstrated in the previous sections, several aspects regarding practical applications, methodological limitations, and computational efficiency deserve further discussion.

From the perspective of engineering applications, the proposed approach provides an efficient tool for the design of acoustic coatings used in underwater vehicles. In practical underwater vehicle design, such as submarines or unmanned underwater vehicles, acoustic coatings are commonly employed to reduce acoustic signatures and improve stealth performance. The ELDNN-based inverse design framework enables rapid identification of structural parameters that satisfy target acoustic performance requirements, which can significantly reduce the design cycle of acoustic coating structures.

Despite the promising performance of the proposed model, several limitations should be acknowledged. First, the training dataset is generated based on theoretical simulations, and although experimental validation has been performed, the number of experimental samples remains limited. Second, the current model mainly focuses on a specific micro-perforated acoustic coating configuration, and its applicability to more complex multilayer acoustic structures requires further investigation. Future work may include expanding the dataset and incorporating more experimental measurements to further improve the model generalization ability.

Compared with existing prediction methods reported in the literature, the proposed ELDNN framework demonstrates improved prediction accuracy and stability. Traditional regression-based approaches often struggle to capture the nonlinear relationship between geometric parameters and acoustic performance, while conventional deep neural networks may suffer from unstable predictions when dealing with complex acoustic parameter spaces. By integrating ensemble learning with deep neural networks, the ELDNN model effectively enhances prediction robustness and reduces the influence of individual model bias.

Another important advantage of the proposed approach lies in its computational efficiency. Traditional optimization methods for acoustic structure design, such as parametric sweeps or evolutionary algorithms, typically require a large number of repeated simulations, which can be computationally expensive. In contrast, once the ELDNN model is trained, the prediction of structural parameters corresponding to target acoustic performance can be obtained almost instantaneously. This significantly reduces computational cost and makes the proposed framework particularly suitable for rapid design and optimization of acoustic structures.

## 7. Conclusions

This study proposes and validates a novel underwater sound absorption structure model by integrating Micro-Perforated Panels (MPP) with acoustic coatings, utilizing an improved transfer function method. The performance of the model is further enhanced by the application of an Ensemble Learning-based Deep Neural Network (ELDNN), which facilitates efficient parameter prediction and optimization. The results demonstrate that MPP-based acoustic coatings significantly improve low-frequency sound absorption, especially in the 50 Hz to 2000 Hz range, offering substantial advantages over traditional acoustic coatings. Through a combination of numerical simulations, experimental validation, and deep learning-driven optimization, the model provides a comprehensive theoretical framework and practical design strategies for underwater acoustic material development and submarine stealth technology. The specific conclusions of this study are as follows:

1. A new underwater sound absorption model based on the transfer function method is proposed and validated through numerical simulations, experimental data, and deep learning predictions. The deep learning framework significantly accelerates the design process and enhances the accuracy of sound absorption predictions, thereby optimizing the model’s performance.
2. The MPP-integrated acoustic coating model (MPPACL) achieves more than 30% improvement in low-frequency sound absorption when compared to conventional acoustic coatings. A distinct absorption peak at 1600 Hz is observed, indicating enhanced performance in the low-frequency range and demonstrating the superiority of MPPACL in underwater applications.
3. Parametric analysis, including factors such as aperture size, porosity, and coating thickness, reveals that adjusting aperture and porosity can effectively improve low-frequency absorption while maintaining high-frequency performance. The proposed ELDNN model, by mining the internal correlation of “structure–acoustics” dual-modal features through deep networks, reduces the prediction error of sound absorption coefficient in the low-frequency range (50–2000 Hz) by 20% compared with traditional single-modal shallow ensemble models. The use of deep learning for optimization allows for more precise predictions, refining the design parameters and further improving the overall acoustic performance of the coating.

Although the proposed ELDNN framework demonstrates promising performance for the inverse design of acoustic structures, several challenges should be considered in practical implementation. In real engineering applications, manufacturing tolerances and material property variations may introduce uncertainties that affect the acoustic performance of the designed structures. For example, small deviations in pore diameter, cavity depth, or rubber material properties may lead to discrepancies between predicted and measured results. In addition, environmental factors such as temperature, pressure, and installation conditions may also influence the acoustic response of underwater acoustic coatings.

Future research will focus on improving the robustness and generalization capability of the proposed model. One possible direction is to expand the dataset by incorporating more complex structural configurations and additional experimental measurements. Furthermore, integrating the ELDNN framework with high-fidelity acoustic simulations and advanced optimization strategies could further enhance the efficiency and applicability of the inverse design process. These developments may provide more reliable tools for the rapid design and optimization of underwater acoustic structures.

## Figures and Tables

**Table 1:** Material Parameters.

Parameters	Value
Rubber density (kg/m^3^)	1100
Young’s modulus of rubber (GPa)	0.14
Loss factor of rubber	0.23
Poisson’s ratio of rubber	0.49
Air density (kg/m^3^)	1.21
Water density (kg/m^3^)	998
Speed of sound in water(m/s)	1483
Density of steel (kg/m^3^)	7800
Poisson’s ratio of steel	0.3
Young’s modulus of steel (GPa)	210

**Table 2:** Parameter Summary.

Category	Parameter	Value
Network architecture	Hidden layers	8
	Neurons per layer	20
	Activation function	ReLU
	Batch normalization	Yes
Regularization	Dropout rate	0.2
	L2 regularization	1 × 10^−4^
Optimization	Optimizer	Adam
	Batch size	128
	Epochs	300
Dataset split	Training set	80%
	Validation set	10%
	Test set	10%
Ensemble strategy	Number of sub-models	8
Learning rate range		1.0005 × 10^−4^–8.818 × 10^−3^
Output fusion	Ensemble method	Average prediction

**Table 3:** Comparison of prediction results for the 400–5000 Hz dataset.

Target Performance Parameter		Geo Metrical Parameter (m)			Predicted Geometric Parameter (m)			Predicted Performance Parameter	
*f* (Hz)	*α*	*a*	*b*	*c*	*a*	*b*	*c*	*f* (Hz)	*α*
450	0.8176	0.0066	0.00015	1	0.006491	0.000164	1.3369	492	0.8467
550	0.7994	0.0066	0.0002	1	0.006342	0.000216	1.2643	546	0.8193
650	0.8407	0.0048	0.0001	2	0.005974	0.000115	1.4503	681	0.8441
750	0.9164	0.0066	0.00015	3	0.006788	0.000181	2.7975	745	0.9316
850	0.9474	0.007	0.0002	4	0.006833	0.000271	3.7615	883	0.9511
950	0.9610	0.0074	0.00055	2	0.006848	0.000554	2.7743	933	0.9644
1000	0.9961	0.007	0.0001	13	0.00722	0.000192	12.329	991	0.9922
2000	0.9480	0.0054	0.0008	13	0.005391	0.000775	12.5934	2016	0.9494
2500	0.9089	0.0044	0.0009	16	0.004391	0.00091	15.8816	2458	0.9102
3000	0.8436	0.0032	0.0007	10	0.003231	0.000707	10.5078	2949	0.8433
3500	0.8125	0.0026	0.0006	13	0.002603	0.000664	12.5381	3452	0.6163
4000	0.7453	0.0014	0.00075	4	0.00133	0.000743	3.7296	4022	0.7521

**Table 4:** Comparison of prediction results for random data.

Target Performance Parameter		Geo Metrical Parameter (m)			Predicted Geometric Parameter (m)			Predicted Performance Parameter	
*f* (Hz)	*α*	*a*	*b*	*c*	*a*	*b*	*c*	*f* (Hz)	*α*
1900	0.9254	0.005	0.0003	13	0.004942	0.00037	13.1727	1937	0.9233
1500	0.9895	0.007	0.0008	9	0.006948	0.00070	10.3622	1531	0.9869
1750	0.9277	0.0052	0.0006	5	0.005134	0.00063	4.2882	1759	0.9168
1900	0.9544	0.0056	0.00085	10	0.005519	0.00087	12.0729	1931	0.9518
1250	0.9188	0.0058	0.0003	4	0.005408	0.00036	3.6223	1243	0.9116
1600	0.9519	0.0058	0.00065	5	0.005642	0.00066	5.4333	1643	0.9450
1650	0.6218	0.0022	0.00015	2	0.002437	0.00012	1.5669	1658	0.6110
2350	0.9051	0.0044	0.0008	9	0.004293	0.00084	10.3547	2389	0.9046
4250	0.7591	0.0016	0.001	10	0.001484	0.00091	10.7206	4280	0.7575
4250	0.7314	0.0012	0.0006	11	0.001161	0.00061	10.6173	4226	0.7340
3350	0.8371	0.003	0.0009	16	0.002957	0.00095	15.6853	3319	0.8385
1600	0.9764	0.0064	0.0007	8	0.006412	0.00064	9.7368	1602	0.9735

**Table 5:** Accuracy summary table.

Prediction Variable	MAE	RMSE	R^2^	Confidence Interval
Frequency (f) (Hz)	26.8 Hz	33.7 Hz	0.998	±18.5 Hz
Absorption coefficient (α)	0.0046	0.0062	0.995	±0.0032
Structural parameter (a) (m)	0.000066	0.000084	0.997	±0.00005
Structural parameter (b) (m)	0.000036	0.000051	0.998	±0.00003
Structural parameter (c)	0.438	0.573	0.991	±0.31

**Table 6:** Comparison of prediction results for the 400–5000 Hz dataset.

Target Performance Parameter		Geo Metrical Parameter (m)			Predicted Geometric Parameter (m)			Predicted Performance Parameter	
*f* (Hz)	*α*	*a*	*b*	*c*	*a*	*b*	*c*	*f* (Hz)	*α*
450	0.9634	0.0068	0.0001	2	0.007154	0.000129	2.9937	452	0.9600
550	0.7944	0.0064	0.0002	1	0.006284	0.000209	1.2635	564	0.8086
650	0.8785	0.007	0.0004	1	0.006663	0.000362	1.3437	704	0.8878
750	0.8210	0.006	0.00035	1	0.005891	0.000375	1.4075	772	0.8278
850	0.8455	0.006	0.00045	1	0.005588	0.000431	1.5270	872	0.8545
950	0.9329	0.0062	0.00015	5	0.006488	0.000165	4.7042	964	0.9421
1000	0.9812	0.0064	0.0001	9	0.007082	0.000189	9.7895	941	0.9804
2000	0.9144	0.0048	0.00095	4	0.004783	0.000909	4.1596	2031	0.9141
2500	0.8848	0.004	0.00055	12	0.004017	0.000519	12.5887	2451	0.8862
3000	0.8147	0.0028	0.00035	11	0.002758	0.000338	10.7344	2908	0.8195
3500	0.7556	0.0018	0.0005	5	0.001692	0.000508	5.1455	3489	0.7598
4000	0.7346	0.0012	0.00035	7	0.001221	0.000368	7.3413	3971	0.7402

**Table 7:** Comparison of prediction results for random data.

Target Performance Parameter		Geo Metrical Parameter (m)			Predicted Geometric Parameter (m)			Predicted Performance Parameter	
*f* (Hz)	*α*	*a*	*b*	*c*	*a*	*b*	*c*	*f* (Hz)	*α*
2700	0.8170	0.003	0.0007	4	0.002838	0.000681	4.4644	2714	0.8132
1400	0.9511	0.006	0.00025	9	0.005851	0.000293	8.6464	1441	0.9488
1350	0.9912	0.0074	0.0009	6	0.007075	0.000859	6.8946	1332	0.9893
1750	0.9569	0.0058	0.00055	9	0.00561	0.000512	9.9875	1746	0.9551
2200	0.8853	0.0042	0.0007	5	0.004063	0.000681	5.129	2239	0.8807
2450	0.8692	0.0038	0.00055	8	0.003683	0.000553	9.0029	2493	0.8664
3950	0.7224	0.001	0.0005	4	0.001135	0.000566	5.2692	3980	0.7209
1950	0.8866	0.0044	0.00085	3	0.004203	0.000821	4.4109	1999	0.8827
4100	0.7717	0.0018	0.00045	14	0.001717	0.000439	13.6956	4121	0.7790
2950	0.7813	0.0024	0.00025	9	0.002319	0.000285	8.3342	2917	0.7793
1500	0.9874	0.007	0.00055	14	0.0069	0.000586	13.3984	1536	0.9876
1350	0.9703	0.0066	0.00095	3	0.006469	0.000974	3.2918	1336	0.9686

**Table 8:** Accuracy summary table.

Prediction Variable	MAE	RMSE	R^2^	Confidence Interval
Frequency (f) (Hz)	30.6 Hz	38.9 Hz	0.997	±21.4 Hz
Absorption coefficient (α)	0.0051	0.0073	0.994	±0.0036
Structural parameter (a) (m)	0.000071	0.000095	0.996	±0.00005
Structural parameter (b) (m)	0.000041	0.000057	0.997	±0.00003
Structural parameter (c)	0.472	0.618	0.989	±0.34

## Data Availability

The original contributions presented in this study are included in the article. Further inquiries can be directed to the corresponding author.
